# Studies of platelet antibodies at MacKay Memorial Hospital in Taiwan: Methods, case reviews and a possible case of post‐transfusion purpura

**DOI:** 10.1111/vox.70200

**Published:** 2026-02-17

**Authors:** Chiang Chen‐Yu, Chang Hsiao‐Lin, Chan Yung‐Shu, Cheng Wern‐Cherng, Lin Marie

**Affiliations:** ^1^ Department of Clinical Laboratory, Blood Bank MacKay Memorial Hospital Taipei City Taiwan

**Keywords:** CD36, history of platelet transfusion, platelet antibodies, post‐transfusion purpura, transfusion refractoriness

## Abstract

**Background and Objectives:**

Platelet antibody screening and identification are crucial and challenging. MacKay Memorial Hospital has been dedicated to this for nearly four decades. We reviewed our experience with platelet antibody detection methods and prevalence, and presented a possible case of post‐transfusion purpura caused by anti‐CD36.

**Materials and Methods:**

We analyzed platelet antibody screening results from 1988 to 2024. We used solid phase red cell adherence test (SPRCA) from 1988 to 2018 (9268 samples), and parallel SPRCA/enzyme‐linked immunosorbent assay (ELISA) from 2019 to 2024 (2037 samples). Positive results are further identified through flow cytometry, monoclonal antibody immobilization of platelet antigens (MAIPA), or in‐house platelet antigen panels. We also studied a possible case of post‐transfusion purpura (PTP) caused by anti‐CD36. A 79‐year‐old woman was treated with numerous platelet transfusions that were ineffective, with generalized purpura appearing a few days later following massive transfusion.

**Results:**

From 1988–2018, 2800 of 9268 samples (30.2%) were positive. Six cases of neonatal alloimmune thrombocytopenia (NAITP) were identified (two anti‐HPA‐3a, two anti‐HPA‐5a, and two anti‐CD36). From 2019–2024, 778 of 2037 samples (38.19%) were positive. Specific antibodies were identified in eight cases, and six of them were anti‐CD36. Introduction of ELISA resulted in a 15‐fold detection rate of anti‐CD36 (0.02% to 0.29%), suggesting previous underestimation. In the possible PTP case, massive transfusion was started on Day 8 of hospitalization, purpura appeared on Day 15, anti‐CD36 was identified on Day 21, and CD36‐negative platelet transfusion was initiated on the following day. However, the patient eventually died of multiorgan failure on Day 29.

**Conclusion:**

The prevalence of anti‐CD36 antibody in the Taiwanese population was previously underestimated. Clinicians should be aware that not only human platelet antigen (HPA) but CD36 should also be considered when platelet refractoriness persists despite HLA‐matched transfusion, as anti‐CD36 may cause serious complications.

**Highlights**

- It is important to use multiple methods simultaneously to enhance the detection of platelet antibodies.
- Anti‐CD36 may be more prevalent than previously thought, and clinicians should consider this possibility when studying platelet transfusion refractoriness.
- Anti‐CD36 may cause post‐transfusion purpura, and CD36‐negative platelets should be given in such cases.

## INTRODUCTION

Platelet antibody screening and identification are crucial yet challenging in diagnosing platelet transfusion refractoriness (PTR), neonatal alloimmune thrombocytopenia (NAITP) and immune thrombocytopenic purpura (ITP).

PTR occurs when platelet increment fails to reflect the transfused numbers. It is caused by immune factors (antibodies against HLA, human platelet antigen [HPA], ABO or CD36) or non‐immune factors (fever, sepsis, bleeding or splenomegaly). In this paper, we examine our institution's historical development of platelet antibody screening methods, present specific cases with identified antibodies and analyse a case of anti‐CD36‐antibody‐related transfusion refractoriness.

## EVOLUTION OF PLATELET ANTIBODY SCREENING METHODS

### Early development (1987–2018)

In 1987, MacKay Memorial Hospital (MMH) established an in‐house solid‐phase red cell adherence test (SPRCA) [1] using platelet‐rich plasma from 12 de‐identified random donors. The programme was officially launched in July 1988 and began accepting requests from other institutions in August 1989. SPRCA served as the main screening method, supported by lymphocytotoxicity test (LCT), monoclonal antibody–specific immobilization of platelet antigens (MAIPA) or SPRCA with chloroquine‐treated platelets for ambiguous results.

### Current methodology (2019–present)

Since 2019, MMH has employed parallel testing with SPRCA and qualitative solid‐phase enzyme‐linked immunosorbent assay (ELISA) (PAKPLUS® assay, Immucor) [2]. A positive result from either test is reported as positive screening. Antibody specificity is determined through additional tests including flow cytometry, MAIPA (with Taiwan Blood Services Foundation assistance) or reactivity with an in‐house platelet antigen panel from MMH colleagues.

## PLATELET ANTIGEN FREQUENCY IN TAIWAN

In the early stages of our screening programme, Lin et al. published research on platelet antigen frequency in the Taiwanese population [3], finding that 100% of participants were positive for HPA‐1a, 9% for HPA‐2b, 77% for HPA‐3a, 100% for HPA‐4a, 0.5% for HPA‐4b and 96% for CD36. Thus, anti‐HPA‐1a would be extremely rare among Taiwanese compared with Whites [4], while highlighting other population‐specific antigen distribution patterns. The publication is believed to be the earliest investigation of platelet antigen frequency among Taiwanese.

## THE IDENTIFICATION CHALLENGE OF HPA‐3a AND HPA‐3b

Between 1988 and 2018, a total of 9268 samples were tested using mainly SPRCA, with 2800 positive samples, a positivity rate of 30.2%. We identified six NAITP cases with specific platelet antibodies: two anti‐HPA‐3a, two anti‐HPA‐5a and two anti‐CD36. Case studies revealed the complex nature of HPA‐3a and ‐3b antibodies [5, 6], with some only reacting with freshly prepared platelets while others with both fresh and stored platelets. This heterogeneity was attributed to a proposed ‘labile component’ on glycoprotein IIb that undergoes structural changes affecting antibody binding [7]. The phenomenon strongly suggests the importance of using multiple methods simultaneously to enhance detection sensitivity.

## DETECTION METHOD DISCREPANCIES (2019–2024)

From January 2019 to June 2024, 2037 samples were tested primarily for ITP and PTR investigation, with 778 samples (38.19%) yielding positive results. Significant discrepancies were observed between detection methods:

1. 326 samples (16%) were positive by both SPRCA and ELISA;
2. 386 samples (18.95%) were positive by ELISA only;
3. 66 samples (3.24%) were positive by SPRCA only;
4. Overall positivity rate: 34.95% for ELISA versus 19.24% for SPRCA.

During this period, eight cases had specific antibodies identified: six anti‐CD36, one anti‐HPA‐5a and one anti‐HPA‐5b. Among the six anti‐CD36 cases, five were MMH patients with PTR, and one was an external NAITP case. Three cases were confirmed as type 1 CD36 deficiency, while the other three cases lacked complete antigen typing. The remaining unidentified samples were mainly from patients with a clinical diagnosis of ITP, and therefore antibody specificity was not identified because of low clinical significance, as management of ITP is independent of antibody specificity.

## CASE PRESENTATION: ANTI‐CD36‐RELATED POST‐TRANSFUSION PURPURA‐LIKE SYNDROME

### Clinical presentation

This case was a 79‐year‐old woman with a previous medical history of left upper lobe lung adenocarcinoma, hypertension, diabetes mellitus, Parkinsonism, left femoral neck fracture and a spontaneous subarachnoid haemorrhage.

The patient initially visited our emergency department for right side chest pain, shortness of breath and cough with whitish sputum. There was no fever, but she had tachycardia and a blood pressure of 125/55 mmHg. Laboratory examination revealed a haemoglobin of 11.2 g/dL, leukocytosis with left shift (white blood cell count 20.5 × 10^3^/μL, band neutrophil 11%) and an elevated C‐reactive protein (34.18 ng/mL). Chest radiograph had increased infiltration of right lower lung field. She was later found to have severe sepsis and septic shock, so she was intubated and admitted to the intensive care unit (ICU) on the same day.

The clinical course was complicated. On Day 8, due to a prolonged prothrombin time and activated partial thromboplastin time (international normalized ratio [INR] = 1.42), 4 units (1 unit equals to 250 mL of whole blood) of fresh frozen plasma (FFP) was administered to correct her coagulation abnormalities. However, the disease progressed, evidenced by anaemia (haemoglobin 5.0 g/dL) and worsening thrombocytopaenia (98,000) and coagulopathy (INR = 2.02) on Day 10. On the same day, she was transfused with 6 units of red blood cells, 4 units of fresh frozen plasma and 1 apheresis unit (PH) of platelets (equivalent to 10 to 12 units of platelet concentrate).

The patient's condition deteriorated critically with gastrointestinal bleeding since Day 11. We found bloody stools of 180, 1330 and 830 mL on Days 11, 13 and 14, respectively.

**FIGURE 1:**
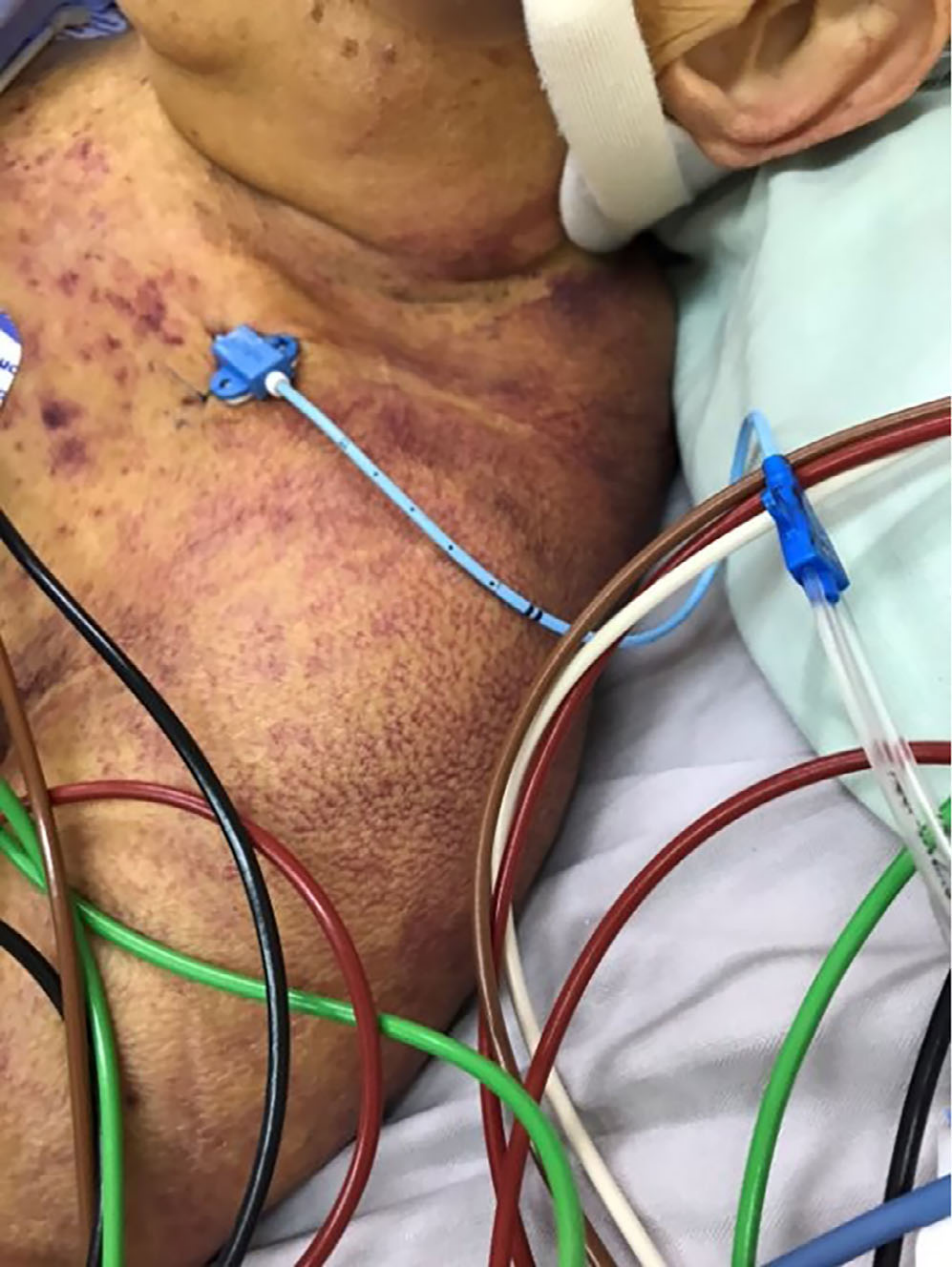
Purpuric appearance of the patient worsening since Day 15 of hospitalization.

**FIGURE 2:**
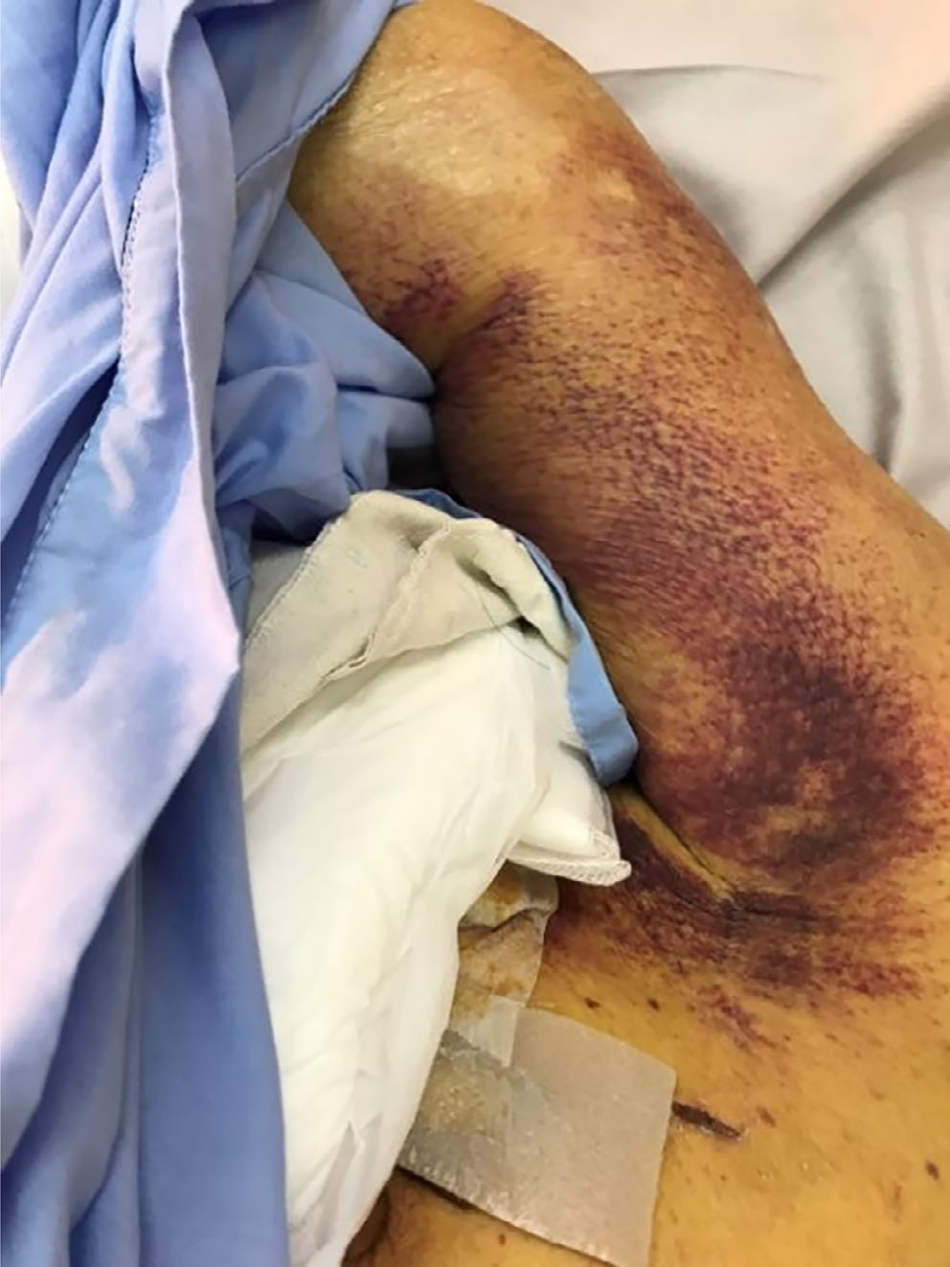
Petechiae on the arm at Day 15.

Despite massive transfusion, the patient's platelet count showed almost no increment, and purpuric skin lesions appeared from Day 15 (shown in Figures 1 and 2). Such refractoriness to platelet transfusion raised concern for immunological problems. However, because of the Taiwanese lunar New Year break, the testing of platelet antibodies was delayed, and anti‐CD36 was not identified until Day 21.

Although the administration of CD36‐negative platelet transfusion was initiated on the next day, it was apparently too late. The patient by then had escalating bilirubin, aspartate aminotransferase, alanine aminotransferase and hepatitis B virus viral load, which indicated a hepatitis B flare‐up. Despite plasma exchange for multiple organ failure and continuous haemodialysis, the patient died on Day 29.

### Laboratory diagnosis

A total of 36 units of red blood cells, 31 apheresis units of platelets (4 of them CD36 negative), 51 units of fresh frozen plasma and 28 units used for plasma exchange, 1 unit of whole blood and 6 units of frozen plasma were transfused altogether.

**FIGURE 3:**
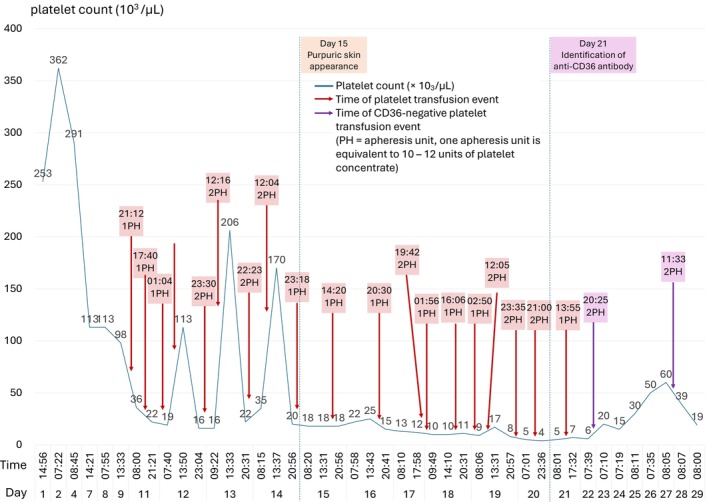
Platelet count and transfusion events during hospitalization. Red arrows indicate transfusion events, and purple arrows are especially CD36‐negative platelet transfusion events. The black arrow at Day 15 indicates onset of purpuric skin lesions and presence of refractoriness. The black arrow at Day 21 indicates confirmation of anti‐CD36 antibodies. Example: On Day 9 of admission, the platelet count was measured as 98 × 10^3^/μL at 13:33, and the patient received 1 apheresis unit (PH) of platelets at 21:12. Note the minimal platelet increment despite aggressive transfusion from Day 15, which demonstrates severe platelet refractoriness.

The platelet count measured during the hospitalization, the quantity of transfused platelets and the platelet count after transfusion are detailed in Figure 3.

Anti‐CD36 was identified using parallel SPRCA and ELISA method, with other antibodies excluded, and was confirmed by MAIPA provided by Taiwan Blood Services Foundation (FA6‐152 monoclonal antibody, Abcam). Optical density (OD) value was 0.245, exceeding the positive cutoff of 0.2. Lastly, CD36 phenotyping was also performed by Taiwan Blood Services Foundation. The result showed platelets negative for CD36. Genotyping could not be performed to identify the specific genetic mutation because no sample had been saved for such testing.

### Differential diagnosis

Before the onset of purpura in the skin, sepsis‐induced thrombocytopaenia and disseminated intravascular coagulation (DIC) may have existed throughout the hospitalization course. None of the medications administered was likely to have contributed to drug‐induced thrombocytopaenia. The timing of massive platelet transfusion (Day 9) and purpuric skin appearance (Day 15) with simultaneous refractoriness strongly supports an alloimmune mechanism rather than simply sepsis or DIC.

This case seems to be a rare post‐transfusion purpuric (PTP) syndrome associated with anti‐CD36. Clinically, it closely resembled PTP, with generalized purpura, severe thrombocytopaenia and ineffective standard platelet transfusions [8]. To our knowledge, this is the first reported case of suspected anti‐CD36‐related PTP‐like syndrome in the Taiwanese population [4].

## DISCUSSION

The frequency of anti‐CD36 in the Taiwanese population appears to have been underestimated during the 1988–2018 period, primarily because of technical limitations of the screening methods employed [9]. Between 1988 and 2018, anti‐CD36 accounted for only two NAITP cases. But following the introduction of ELISA in 2019, they rapidly became the most frequently detected specific antibody, with a 15‐fold increase in annual detection rate. The dramatic shift despite the same population of patients (ITP and PTR) once again highlights the underestimation of anti‐CD36.

This discrepancy can have resulted from several methodological factors. SPRCA uses intact platelets. Thus, membrane structures may mask the region that binds to anti‐CD36 [7]. Additionally, the monoclonal antibody FA6‐152 used in MAIPA targets the same region of CD36 that patient antibodies often recognize, potentially resulting in competitive inhibition and false‐negative results [9, 10]. The increase in anti‐CD36 detection after ELISA implementation suggests that this antibody may be the most prevalent specific platelet antibody in the Taiwanese population after HLA antibodies.

The higher positive rate of ELISA (34.95%) compared to SPRCA (19.24%) can be attributed to ELISA's use of extracted platelet glycoproteins, allowing greater antigen exposure. Conversely, the small percentage of SPRCA‐positive but ELISA‐negative results (3.24%) may be explained by ELISA's limited coverage of HPA antigens (missing HPA‐6 through HPA‐21) and potential differences in HLA antigen distribution between the White‐derived ELISA materials and Asian populations [4, 11, 12], as well as different pooled donors from different lot numbers, which may cause missed detection of specific HLA antibodies.

This study has significant implications for transfusion medicine in Taiwan. The recognition that anti‐CD36 may be more prevalent than previously thought should inform screening protocols and management strategies for PTR. For refractory patients, after HLA‐matched platelets prove ineffective, screening for anti‐CD36 and other HPA antibodies should be considered, particularly in our population, where CD36 deficiency prevalence is known.

In conclusion, platelet antibody screening remains challenging and deserves continued attention. Our experience highlights the heterogeneity of HPA‐3a and HPA‐3b antibodies and the previous underestimation of anti‐CD36 prevalence in the Taiwanese population. We emphasize the importance of comprehensive antibody screening in cases of PTR and recommend considering CD36 status when HLA‐matched transfusions are ineffective.

The case of suspected anti‐CD36‐related post‐transfusion purpura demonstrates the potential clinical severity of these antibodies and suggests that CD36‐negative platelet transfusions should be considered in such cases. Further efforts could be made by clinicians to enhance the safety and appropriateness of blood transfusion.

## CONFLICT OF INTEREST STATEMENT

The authors declare no conflicts of interest.

## Data Availability

The data that support the findings of this study are available from the corresponding author upon reasonable request.
